# The Application of Green-Synthesis-Derived Carbon Quantum Dots to Bioimaging and the Analysis of Mercury(II)

**DOI:** 10.1155/2019/8183134

**Published:** 2019-11-30

**Authors:** Qianchun Zhang, Xiaolan Zhang, Linchun Bao, Yun Wu, Li Jiang, Yuguo Zheng, Yuan Wang, Yafei Chen

**Affiliations:** ^1^Key Laboratory of Chemical Synthesis and Environmental Pollution Control-Remediation Technology of Guizhou Province, School of Biology and Chemistry, Xingyi Normal University for Nationalities, Xingyi 562400, China; ^2^Clinical Laboratory, Qian Xi Nan People's Hospital, Xingyi 562400, China

## Abstract

Ginkgo leaves were used as precursors for the hydrothermal synthesis of carbon quantum dots (CQDs), which were subsequently characterized by transmission electron microscopy as well as Fourier-transform infrared, X-ray powder diffraction, and X-ray photoelectron spectroscopy. The prepared CQDs exhibited a fluorescence quantum yield of 11% and superior water solubility and fluorescence stability, as well as low cytotoxicities and excellent biocompatibilities with A549 and HeLa cells; these CQDs were also used to bioimage HeLa cells. Moreover, owing to the experimental observation that Hg^2+^ quenches the fluorescence of the CQDs in a specific and sensitive manner, we developed a method for the detection of Hg^2+^ using this fluorescence sensor. The sensor exhibited a linear range for Hg^2+^ of 0.50–20 *μ*M, with an excellent coefficient of determination (*R*^2^ = 0.9966) and limit of detection (12.4 nM). In practice, the proposed method was shown to be highly selective and sensitive for the monitoring of Hg^2+^ in lake water and serum samples.

## 1. Introduction

Carbon quantum dots (CQDs) are receiving significant attention because of their stable photoluminescence properties, lack of bleaching, low toxicity, and biocompatibility [1–3]. These superior qualities are promising for bioimaging applications [4, 5], metal-ion detection [6, 7], fluorescence labelling [8], sensing [9], catalysis [10], and optics [11], with analysis techniques based on CQDs expected to be cost effective, highly sensitive, and easy to use. Current research is directed toward a variety of CQD-preparation methods, such as hydrothermal, microwave-assisted, electrochemical oxidation, chemical oxidation, laser-engraving, and ultrasonic methods, among others, with hydrothermal methods that use cheap and eco-friendly biomass and are also simple, green, and economical, being favoured by researchers.

Nature provides unlimited resources, which provides researchers with the freedom and inspiration to propose new ideas and methods for developing quantum dots with new structures and properties that are less impacting on the environment. Recently, green methods for preparing carbon quantum dots from natural precursors have received extensive attention. Sahu et al. [12] and Zhao et al. [13] used orange juice and garlic as carbon sources to hydrothermally prepare highly biocompatible CQDs with low toxicities; these CQDs were successfully used in bioimaging to good effect. Mercury(II) is an extremely toxic heavy metal pollutant due to its severe risks for human health, and developing efficient detection of trace mercury(II) is very significant for protecting human health. The fluorescence method is more widely applied, and it has avoidably complicated sample preparation, using expensive instrumentations and professional skills. In this regard, some works have been reported for mercury (II) detection [14–17], such as Yue et al. [16] who used apple juice as the carbon source. CQDs with good water solubilities and high stabilities were hydrothermally prepared and used for the highly sensitive and selective detection of mercury (II). Nitrogen and sulfur codoped carbon dots were synthesized by hydrothermal method, and this probe was applied for analysis of mercury (II) in environmental water samples [17]. The CQDS has a significant important function because it determines the selectivity and sensitivity. Therefore, it is important to develop a dependable CQDS for selective and sensitive detection of low concentration levels of mercury (II).

In this study, we used eco-friendly ginkgo leaves as the carbon source. By studying the preparation conditions in detail, we prepared low-cost, biocompatible, and highly blue-fluorescent CQDs using a one-step hydrothermal method. The developed methodology avoids metal contamination and the use of concentrated acids and uses green carbon-, oxygen-, and nitrogen-containing precursors. The prepared CQDs exhibit good membrane permeabilities and excellent biocompatibilities and were used to image HeLa cells. Moreover, the present system was further applied to the rapid and sensitive analysis of mercury(II) in lake water and serum samples, with good recoveries.

## 2. Materials and Methods

### 2.1. Reagents and Materials

3-(4,5-Dimethyl-2-thiazolyl)-2,5-diphenyl-2-H-tetrazolium bromide (MTT) and Dulbecco's modified Eagle medium (DMEM) were obtained from the Suo Laibao Technology Co., Ltd. (Beijing, China). Phosphate-buffered saline (PBS), quinine sulfate dihydrate, Cr(NO_3_)_3_·9H_2_O, Cd(NO_3_)_2_·4H_2_O, MgCl_2_·6H_2_O, Zn(NO_3_)_2_·6H_2_O, CaCl_2_, BaCl_2_·2H_2_O, LiCl, Co(NO_3_)_2_·6H_2_O, MnSO_4_·H_2_O, AgNO_3_, RbCl, PbCl_2_, Fe(NO_3_)_3_.9H_2_O, and FeSO_4_·7H_2_O were purchased from Aladdin (Shanghai, China). Mercury standard solution (100 *μ*g/mL) was purchased from the National Standard Center (Beijing, China). The mercury standard stock solution was diluted stepwise with 10% (v/v) HCl to prepare standard solutions. Isopropanol, NaCl, KCl, SrCl_2_·6H_2_O, AlCl_3_·6H_2_O, Cu(NO_3_)_2_·3H_2_O, and Ni(NO_3_)_2_·6H_2_O were obtained from the Sinopharm Chemical Reagent Co., Ltd. (Beijing, China). All other chemical reagents were of analytical grade. Ginkgo leaves were collected from the Xingyi Normal University for Nationalities on September 16 and blended. A549 cells, HeLa cells, and serum samples were provided by the Qian Xi Nan People's Hospital; ultrapure water (18.2 MΩ cm) was prepared using a Milli-Q gradient A10 system (Millipore, UK) and was used in all experiments.

### 2.2. Instrumentation and Characterization

High-resolution transmission electron microscopy (HRTEM) was performed using a Hitachi-F20 microscope at an accelerator voltage of 200 kV. Fluorescence spectroscopy was conducted on an RF-6000 spectrofluorometer (Shimadzu, Japan). X-ray diffraction (XRD) was carried out using a Bruker AXS D8ADVANCE X-ray diffractometer. X-ray photoelectron spectroscopy (XPS) was performed using an ESCALAB 250Xi (Thermo Scientific, USA) instrument and Fourier-transform infrared (FT-IR) were acquired on a Nicolet 6700 FT-IR spectrometer using KBr pellets. UV-Vis absorption spectra were recorded on a 4501S UV-Vis spectrophotometer (Gangdong, Tianjin, China), and an AFS-9800 instrument was used for hydride-generation atomic fluorescence spectrophotometry (Haiguang, Beijing). A KT7-900-434 high-speed centrifuge was used from Heller International Trading Co., Ltd (Kenda, Germany). Cells were imaged using an IX71-F22FL/fluorescence-inverted microscope (Olympus, Japan), a KQ2200E ultrasonic cleaning machine was used (Kunshan Ultrasonic Instrument, Jiangsu, China), and an AL104 electronic balance from the Mettler Toledo Instrument Co, Ltd. (Shanghai, China) was used. Optical densities were measured using a Multiskan FC ELISA instrument (Thermo Scientific, USA).

### 2.3. Preparing the Carbon Quantum Dots

CQDs were synthesized by a facile hydrothermal method. Briefly, 12.60 g of crushed ginkgo leaves was dispersed in 30 mL of ultrapure water and subjected to ultrasonic oscillation for 30 min, after which the mixture was transferred into a 50 mL Para polyphenyl equipped stainless-steel autoclave and heated at 220°C for 10 h. The reaction mixture was cooled to room temperature, sonicated for 30 min, and then continuously centrifuged at 8000 rpm for 15 min. The resultant transparent yellow solution was filtered through a 0.22 *μ*m membrane filter. The as-prepared CQD solution was diluted by a factor of 10 with ultrapure water and stored at 4°C for further use.

### 2.4. Carbon Quantum Dot Cytotoxicity and Bioimaging

Toxicity testing was carried using the MTT method with A549 and HeLa cells, as follows: A549 and HeLa cells were seeded in the wells of a 96-well plate (about 4 × 10^3^ cells per well) and incubated for 24 h (37°C, 5% CO_2_). The cell culture solution was then removed from each well and replaced with 100 *μ*L of DMEM containing various concentrations of CQDs (0–1000 *μ*g/mL) for 48 h. The cells were then washed, and 20 *μ*L of 5 mg/mL MTT was added to the flat floor of each well to continue culturing. The cell culture solution and MTT were then aspirated and washed with PBS, after which isopropanol was added, the culture was shaken at 100 rpm for 10 min, and the optical density of each well was measured using a 450 nm wavelength enzyme-linked immunosorbent assay to determine cell activity. Control experiments devoid of CQDs were also performed. Each experiment was conducted five times, with the data averaged.

The biolabeling potential of the CQDs was preliminarily evaluated by the cellular imaging of HeLa cells. Firstly, 100 *μ*L of fresh 10% (v/v) PBS/DMEM and HeLa cells (1 × 10^5^/dish) were added to a culture dish and cultured for 24 h (37°C, 5% CO_2_). The CQD solution was then added to the culture dish and allowed to stand for 4 h (37°C, 5% CO_2_). Finally, the cell culture solution was washed three times with PBS solution (10 mM, pH 7.4) and imaged at 405 nm, 488 nm, and 610 nm.

### 2.5. Detecting Mercury Ions

All photoluminescence spectra were recorded on the abovementioned fluorescence spectrophotometer at a fixed excitation wavelength of 380 nm and the emission wavelength 442 nm. In a typical Hg^2+^-detection procedure, 100 *μ*L of CQDs (2.0 mg/mL), 875 *μ*L of PBS buffer (100 mM, pH 7.0), and a series of freshly prepared 25 *μ*L Hg^2+^ samples were added to microcentrifuge tubes. The mixtures were then shaken and incubated for 10 min. The CQD fluorescence-quenching extent is given by *F*/*F*_0_, where *F*_0_ is the fluorescence intensity of the solution in the absence of Hg^2+^, and *F* is the fluorescence intensity after adding Hg^2+^. All experiments were performed at room temperature.

#### 2.5.1. Analysis of Samples

Serum samples (100 *μ*L) from the Qian Xi Nan People's Hospital were accurately measured and added to 10 mL centrifuge tubes, after which 5 mL of water was added to each centrifuge tube. The mixtures were ultrasonicated for 10 min. In addition, water from Wanfeng Lake (Guizhou, China) was also analyzed. All as-prepared water samples were centrifuged at 15000 rpm for 10 min to remove particles, after which they were filtered through a 0.22 *μ*m filter in order to remove suspended impurities. The pretreated water samples were spiked with ultrapure water or different concentrations of standard Hg^2+^ solutions, after which they were analyzed as described above.

## 3. Results and Discussion

### 3.1. Carbon Quantum Dot Preparation Conditions

**Figure 1:**
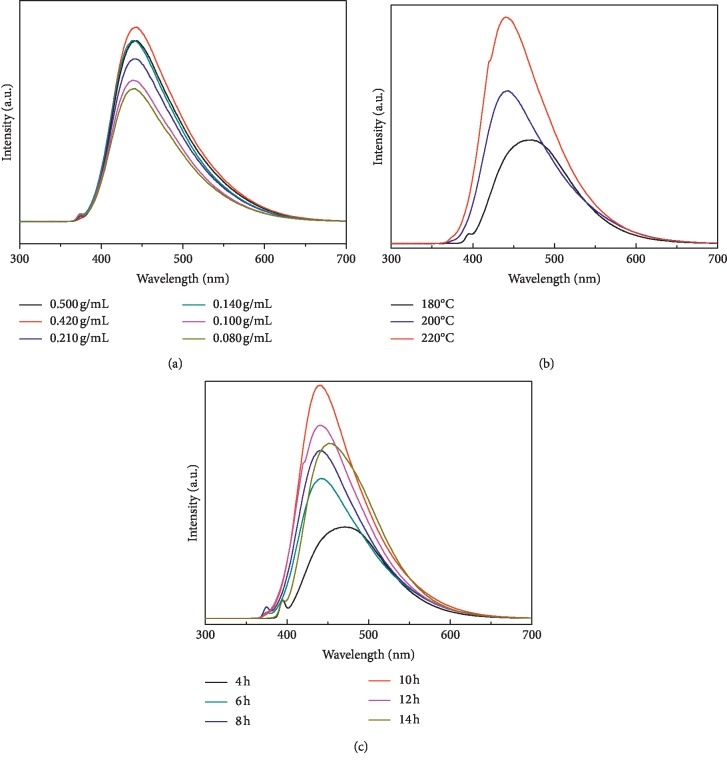
Optimizing the preparation conditions: (a) concentration, (b) reaction temperature, and (c) reaction time.

We first examined concentrations, temperatures, and reaction times as preparation conditions, the results of which are shown in Figure 1. The photoluminescence intensity depends on the number of particles excited. The experimental results showed the prepared CQDs exhibit more particles at a concentration of 0.420 g/mL (Figure 1(a)). The effect of temperature in the range of 180–220°C was also evaluated (Figure 1(b)). When compared with 180°C, higher temperature resulted in a rather large increase in photoluminescence intensity. This is because the increase in temperature enhanced the interaction between the precursor and water solution. Considering the operating security of para-polyphenyl equipped stainless-steel autoclave, the temperature of 220°C was used. The effect of reaction time was also investigated at a concentration of 0.420 g/mL, from 4 h to 14 h at a constant temperature of 220°C, and the results indicated more CQD particles were obtained and suggested reaction time of 10 h was adopted (Figure 1(c)).

### 3.2. Characterizing the Carbon Quantum Dots

**Figure 2:**
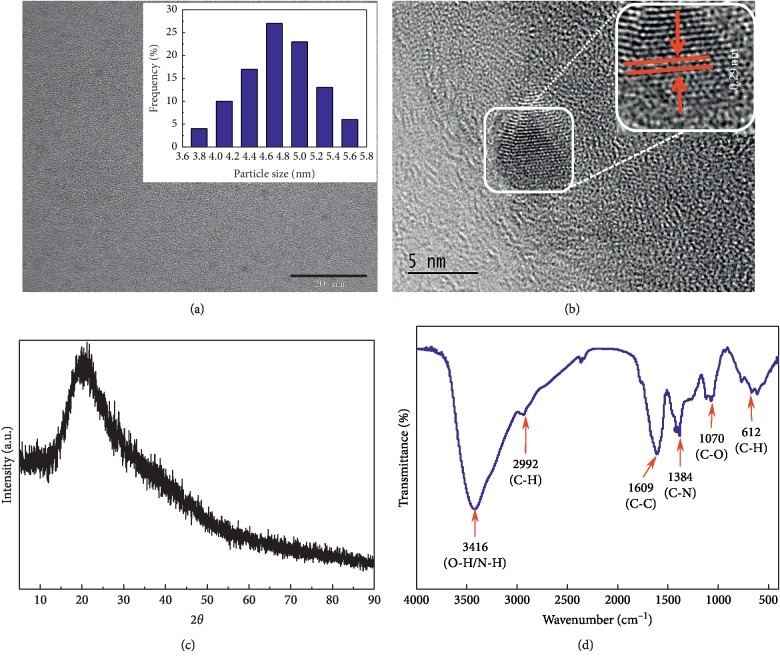
(a) TEM image of the prepared CQDs. Inset shows the size distribution of CQDs. (b) HRTEM reveals lattice spacing of CQDs. (c) XRD. (d) FT-IR spectrum of the CQDs.

The morphology, fine structure, and particle size of the as-prepared CQDs were characterized by HRTEM, as shown in Figure 2(a), which reveals that the CQDs are nearly spherical and monodispersed. Inset in Figure 2(a) shows that the average particle size is about 4.90 nm, with a narrow 3.8–5.8 nm size distribution. HRTEM images (Figure 2(b)) showed that the average lattice spacing of the CQDs was 0.29 nm. The broad XRD peak showed at 2*θ* = 20.6° (Figure 2(c)), suggesting the formation of the 002 Bragg reflections and this result is in good agreement with that reported by Liu et al. [18]. This result confirmed that the as-prepared CQDs is amorphous graphite. These results indicate that the carbon source in the ginkgo leaves had been effectively stripped and converted into CQDs.

FT-IR spectroscopy and XPS were used to determine the functionalization of the CQDs and their chemical compositions. The FT-IR spectrum shown in Figure 2(d) reveals a peak at 3416 cm^−1^ that corresponds to O-H and N-H stretching vibrations, a peak at 2992 cm^−1^ due to C-H stretching vibrations, a C-C stretching peak at 1609 cm^−1^, and a C-N stretching peak at 1384 cm^−1^, while the peaks at 1070 and 612 cm^−1^ are attributed to C-O stretches and out-of-plane C-H bending vibrations. The FT-IR results show that the surfaces of the CQDs contain two hydrophilic functional groups: -OH and -NH2.

**Figure 3:**
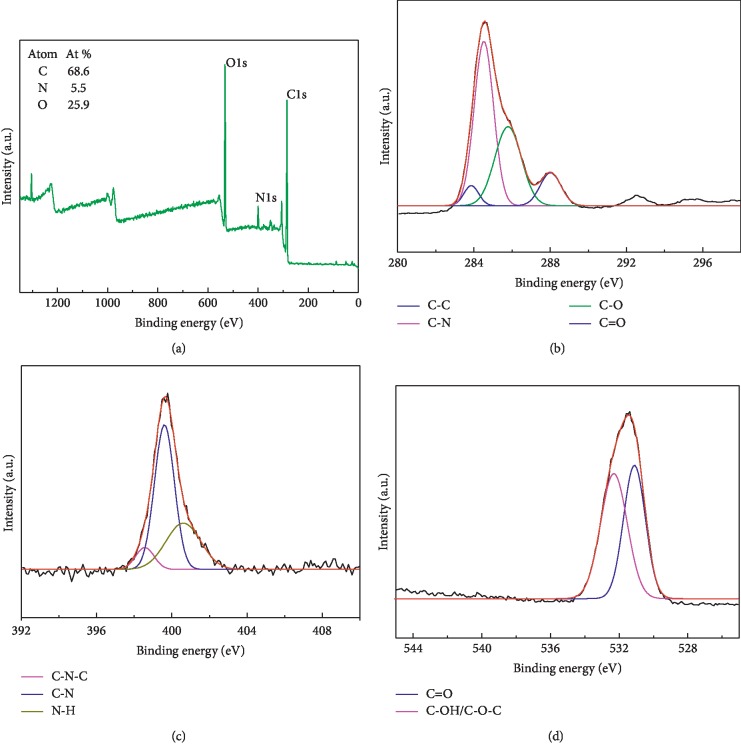
(a) XPS survey spectrum of the CQDs, and the corresponding high-resolution (b) C1s, (c) N1s, and (d) O1s spectra.

These FT-IR assignments were further verified by XPS.  Figure 3(a) shows that the CQDs mainly contain elemental C, N, and O, with bond energies of 284.58, 399.66, and 531.55 eV, respectively; the contents of these elements were determined to be 68.6%, 5.5%, and 25.9%, respectively. The high-resolution C1s XPS spectrum exhibits characteristic peaks at 284.39, 284.48, 286.38, and 288.58 eV (Figure 3(b)), which correspond to C-C, C-N, C-O, and C=O bonds [19], respectively.  Figure 3(c) displays the N1s spectrum, which exhibits peaks that correspond to bond energies of 398.98, 400.01, and 401.68 eV and are due to C-N-C, C-N, and N-H bonds [20], respectively. The O1s spectrum displayed in Figure 3(d) shows bond energies of 531.38 eV and 532.36 eV that correspond to C=O and C-OH/C-O-C bonds [21], respectively. The FT-IR and XPS results reveal that the surfaces of the CQDs contain hydrophilic -COOH, -OH, and -NH2 functional groups that help the CQDs become uniformly dispersed in aqueous solutions, which is beneficial for biological imaging and the detection of Hg^2+^.

### 3.3. Photoluminescence Properties of the CQDs

**Figure 4:**
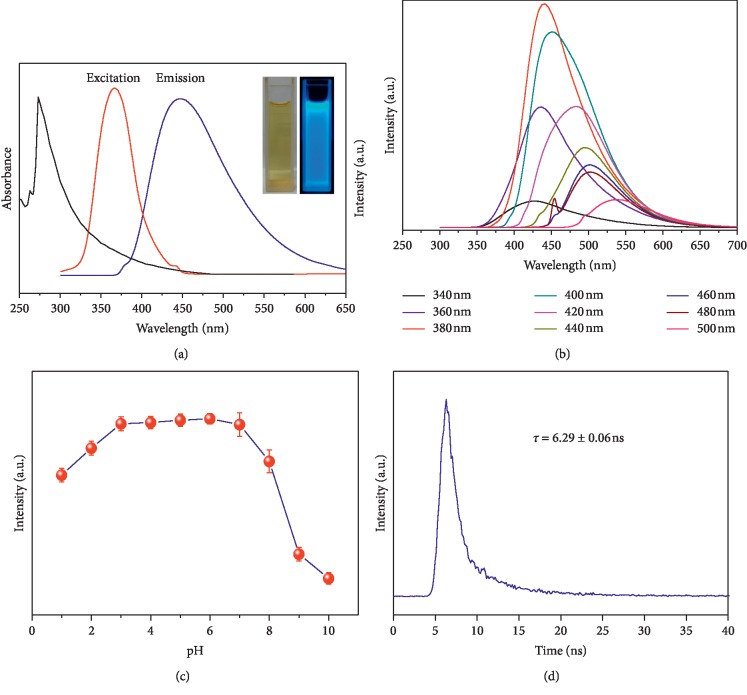
(a) UV-Vis absorption and photoluminescence spectra of CQDs. Inset: images of the N-CD aqueous solution in sunlight (left) and ultraviolet radiation (right). (b) Fluorescence emission spectra of the CQDs excited at various wavelengths. (c) The effect of pH on the fluorescence intensity of the CQDs excited at 380 nm. (d) The fluorescence decay curve of CQDs.

The CQDs exhibit excitation-dependent photoluminescence behaviour commonly observed for fluorescent carbon materials. Fortunately, excitation-dependent photoluminescence behaviour is useful for multicolour imaging applications. The UV-Vis absorption spectrum in Figure 4(a) has an obvious absorption peak at 273 nm, attributed to *π*−*π*^*∗*^ due to C=C functional groups and *n*−*π*^*∗*^ due to C=O functional groups [22]. The fluorescence properties are important, and the result showed very high emission at 442 nm and excitation at 380 nm from CQDs. The CQDs were excited at wavelengths between 340 nm and 500 nm, as shown in Figure 4(b), with the corresponding fluorescence emission maximum observed to increase in intensity, from 928 to 7869 a.u.; the strongest emission was observed when the CQDs were excited at 380 nm, which was determined to be the optimal excitation wavelength. This is ascribable to *π*-*π*^*∗*^ transitions involving the C=C bonds of the CQDs [23]. The fluorescence emission spectrum obtained when excited at 380 nm reveals an emission maximum at about 442 nm, with the emission band covering the entire blue region of the spectrum (420–550 nm). The QY of the as-prepared CQDs in aqueous solution at room temperature was determined to be 11% using quinine sulfate as the reference (QY = 54%).

The surface groups of the CQDs are affected by pH, which is expected to significantly impact the fluorescence intensity. The fluorescence intensity of the as-prepared CQDs when excited at 380 nm is affected little as the pH is varied between 3.0 and 7.0. On the other hand, varying the pH between 1.0 and 3.0 was observed to affect the emission intensity, and it was significantly lower above pH 8.0 (Figure 4(c)). Therefore, the pH should be controlled as much as possible to remain between 3.0 and 7.0 for application purposes. Because the CQD particles are small and can easily agglomerate, the fluorescence intensity is expected to decrease over long periods of time. In our experiment, the as-prepared CQDs exhibited a certain amount of fluorescence attenuation over 300 min; however, the degree of attenuation observed does not affect its use. As shown in Figure 4(d), the decay lifetime of the CQDs was measured and the average fluorescence lifetime for the CQDs was 6.29 ± 0.06 ns.

### 3.4. Carbon Quantum Dot Toxicity and Imaging

**Figure 5:**
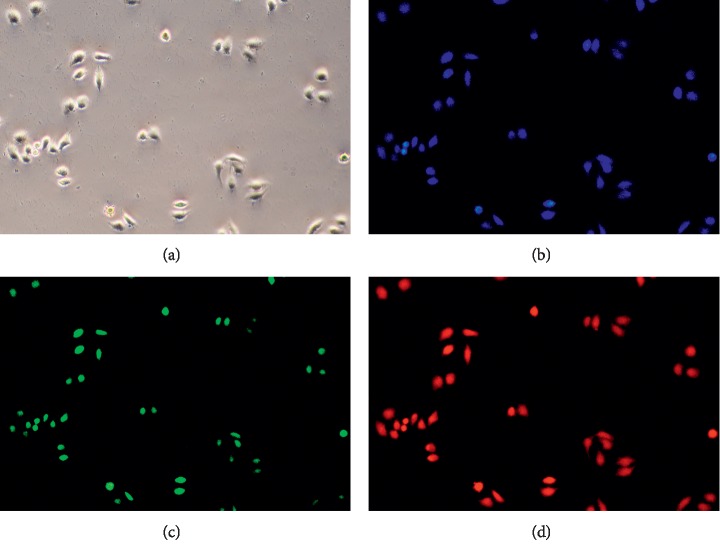
Images of HeLa cells labelled with CQDs. (a) Bright field. Excited cells observed at (b) 405 nm, (c) 488 nm, and (d) 610 nm.

To assess CQD cytotoxicity, the viabilities of A549 and HeLa cells treated with CQDs were determined using the MTT assay. As shown in Figure 5, A549 and HeLa cells were incubated with different doses of CQDs for 48 h, and A549 and HeLa cells were viable more than 95% at concentrations below 100 *μ*g/mL, and DMEA at high concentrations of CQDs at 1000 *μ*g/mL. After culturing for 48 h, these cells were still 82% viable. These results indicated that the prepared CQDs exhibit low toxicity and can be used as biomarkers. HeLa cells were then treated with 50 *μ*g/mL CQDs and observed by fluorescence inversion microscopy at 405 nm, 488 nm, and 610 nm; the cells showed bright blue, red, and green, respectively, at these wavelengths, demonstrating that these CQDs have good permeabilities and biocompatibilities for biological imaging purposes.

### 3.5. Detecting Hg^2+^

**Figure 6:**
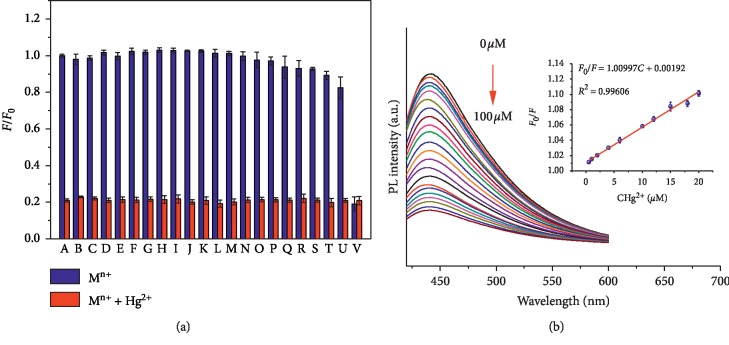
(a) Fluorescence intensities (*F*/*F*_0_) in the presence of 100 *μ*M concentrations of various metal ions before (blue bars) and after (red bars) mixing with 100 *μ*M Hg^2+^. (b) Fluorescence spectra of the CQD system in the presence of different concentrations of Hg^2+^ (excited at 380 nm). Inset: the dependence of *F*_0_/*F* on concentration (0.500–20.0 *μ*M). In (a), (A) blank; (B) Cr^3+^; (C) Cd^2+^; (D) Sr^2+^; (E) Al^3+^; (F) Mg^2+^; (G) Zn^2+^; (H) Ca^2+^; (I) K^+^; (J) Ba^2+^; (K) Na^+^; (L) Li^+^; (M) Co^2+^; (N) Ni^2+^; (O) Mn^2+^; (P) Ag^+^; (Q) Rb^+^; (R) Pb^2+^; (S) Fe^3+^; (T) Cu^2+^; (U) Fe^2+^; (V) Hg^2+^.

We examined the CQD photoluminescence intensity in the presence of 100 *μ*M of representative metal ions, including Cr^3+^, Cd^2+^, Sr^2+^, Al^3+^, Mg^2+^, Zn^2+^, Ca^2+^, K^+^, Ba^2+^, Na^+^, Li^+^, Co^2+^, Ni^2+^, Mn^2+^, Ag^+^, Rb^+^, Pb^2+^, Fe^3+^, Cu^2+^, Fe^2+^, and Hg^2+^. As shown in Figure 6(a), metal ions other than mercury (II), did not obviously change the fluorescence signal when excited at 380 nm, compared to that of the blank. However, the presence of Hg^2+^ ions results in a clear decrease in photoluminescence intensity, which shows that the CQD fluorescence system is very selective and is effectively quenched by Hg^2+^. The various effects of Hg^2+^ at concentration from 0 to 100 *μ*M were further studied, as shown in Figure 6(b), and the fluorescence is clearly quenched in a concentration-dependent manner with increasing levels of Hg^2+^ (Figure 6(b), inset). The linear range for Hg^2+^ was determined to be 0.50–20 *μ*M; with the relationship expressed by the linear equation (*F*_0_/*F* = 0.00463C + 1.01099), good linearity with a coefficient of determination (*R*^2^) of 0.9966 was observed, and the detection limit was determined to be 12.4 nM (*S*/*N* = 3, *n* = 5). The developed method provides excellent sensitivity with Hg^2+^, such as the sensor technique adopted by Noor et al. [24] and by Kamali et al. [25], and the sensor can detect Hg^2+^ with LODs of 590 nM and 338 nM, respectively.

The practical use of the CQDs was further assessed by the specific detection of Hg^2+^ in lake water and serum samples, the results of which were summarized in Table 1. The recoveries for the two sample sets ranged from 89.5% to 103%, with relative standard deviations of less than 4.8%. This method provides results for the determination of Hg^2+^ that were consistent with those obtained by hydride-generation atomic fluorescence spectrophotometry. These results demonstrated that the method is highly accurate and can sensitively and conveniently analyze Hg^2+^ in real samples.

## 4. Conclusions

In this study, we introduced a facile, green, and high-output hydrothermal synthesis procedure for the fabrication of CQDs that are water-soluble and exhibit excellent water dispersibilities and low toxicities. The CQDs exhibited a strong blue fluorescence emission with a QY of 11%. The CQDs are spherical and monodispersed and have a narrow size distribution (average diameter: 4.9 ± 0.90 nm). The CQDs can be used as a novel fluorescence probe that enables both HeLa-cell imaging and sensitive Hg^2+^ detection. The developed method demonstrated good linearity (*R*^2^ = 0.9966), a satisfactory detection limit (12.4 nM), and repeatability (<4.8%) and was shown to be simple, rapid, convenient, and reliable for determining Hg^2+^ in environmental water and serum samples.

## Figures and Tables

**Table 1:** Detecting Hg^2+^ in real samples (*n* = 5).

Sample	Added (*μ*M)	Found (*μ*M)	Recovery (%)	RSD (%)
Lake water	1.00	0.945 (0.967^a^)	94.5	2.5
	10.0	9.61	103	2.1

Serum	1.00	0.895 (0.932^a^)	89.5	4.8
	10.0	9.37	93.7	3.6

^a^The concentrations in parentheses were determined by hydride-generation atomic fluorescence spectrophotometry.

## Data Availability

The data used to support the findings of this study are available from the corresponding author upon request.
